# Comparison of antibacterial activity of alexidine alone or as a final irrigant with sodium hypochlorite and chlorhexidine

**DOI:** 10.1038/bdjopen.2018.3

**Published:** 2018-06-01

**Authors:** Thaís M da Silva, Flávio RF Alves, Márcia TS Lutterbach, Maurício M Paiva, Dennis de Carvalho Ferreira

**Affiliations:** 1 Estácio de Sá University, Endodontics 580, Alfredo Baltazar da Silveira, Rio de Janeiro, Brazil; 2 Endodontics, Rio de Janeiro, Brazil; 3 Microbiology, Rio de Janeiro, Brazil; 4 Electronic Microscopy, Rio de Janeiro, Brazil; 5 Department of Medical Microbiology, Institute of Microbiology, Rio de Janeiro, Brazil

## Abstract

**Aims:**

To compare the antibacterial activity of alexidine (ALX) alone or as a final irrigant in combination with sodium hypochlorite (NaOCl), with the most common canal irrigants, NaOCl and chlorhexidine (CHX).

**Materials and methods:**

Ninety-four root fragments from extracted human teeth were infected with *Enterococcus faecalis* for 24 h and then distributed into 4 groups of 20 fragments each. The NaOCl, CHX and ALX groups were immersed in 1 ml of 2.5% NaOCl, 2% CHX, and 1% ALX for 10 min, respectively. The samples of the NaOCl+ALX group were immersed in 1 ml of 2.5% NaOCl for 10 min followed by 1% ALX for 10 min. Bacteriological samples were taken, cultured, and the colony-forming units were counted.

**Results:**

There was no significant differences among the experimental groups (*P*>0.05) except for the comparisons CHX versus ALX and NaOCl+ALX versus ALX (*P*=0.004). ALX alone was the worst irrigant. CHX and NaOCl+ALX eradicated all bacteria. All experimental groups were significantly more effective than the control group immersed in saline (*P*<0.05).

**Conclusions:**

The antibacterial effect of ALX alone was inferior to 2% CHX and 2.5% NaOCl. However, the combination of NaOCl with ALX as a final irrigant eradicated the biofilms.

## Introduction

The aim of treatment in infected root canals is to eliminate microorganisms from the root canal system and to prevent its reinfection. Bacteria are the main microorganism implicated in the apical periodontitis.^1^ Among them, *Enterococcus faecalis* deserves attention because of its high prevalence in the different types of endodontic infection, especially in persistent infections.^2,3^ The inherent ability of *E. faecalis* to adhere and invade dentinal tubules and form communities in an organized biofilm may contribute to its resistance to irrigant solutions and intracanal medicaments.^4^ Consequently, this microorganism is often chosen to induce *ex vivo* bacterial biofilms in assays comparing antimicrobial solutions.

During the root canal treatment, mechanical debridement is of utmost importance to remove microorganisms and organic content that might serve as nutrients for residual bacteria. Nonetheless, studies have demonstrated that although instrumentation and irrigation are effective in substantially reducing the number of bacteria in infected canals, in many cases bacteria remain in the main root canal even when sodium hypochlorite (NaOCl) is used as the irrigant.^5^ NaOCl is the most common root canal irrigant due to its tissue-dissolving capability, its broad antimicrobial action, as well as its ability to neutralize toxic products.^6,7^ However, NaOCl has many disadvantages, including cytotoxicity, reduced efficacy in the presence of organic matter, and interference with pulp regeneration procedures.^8–10^ These limitations stimulate the search for safer and more effective irrigants. An alternative to NaOCl is chlorhexidine digluconate (CHX). This irrigant is a bisbiguanide disinfectant that has high antimicrobial activity, substantivity, and biocompatibility. However, CHX has been shown to have no tissue-dissolving activity and, when combined with NaOCl, produces para-chloroaniline, a toxic precipitate.^11–13^

The search for the ideal root canal irrigant revealed another candidate - alexidine (ALX). This substance is a bisbiguanide disinfectant similar to CHX, it contains two hydrophobic ethylhexyl groups in its structure and it has a higher affinity for major bacterial virulence factors such as bacterial lipopolysaccharide and lipoteichoic acid than CHX.^14,15^ Alexidine is used as a disinfectant in contact lens solutions^16,17^ and as an antiseptic in mouthwashes.^18–20^ A recent study showed that the antibacterial activity of alexidine against *E. faecalis* infecting dentin blocks was superior to CHX.^21^ Also, while there are many reports of allergic reactions, including anaphylaxis, following exposure to chlorhexidine, there is a lack of reports for ALX.^22–24^ Another important advantage of alexidine is that its combination with NaOCl does not produce any precipitate or para-chloroaniline.^25^ Therefore, the combination of NaOCl as the main irrigant with ALX as the final irrigant may be of great utility for the treatment of endodontic infections.

The purpose of this study was to compare the efficacy of ALX alone or as a final irrigant in combination with NaOCl with the most common canal irrigants, NaOCl, and chlorhexidine

## Materials and methods

### Preparation of dentin blocks

Forty-seven upper canines were obtained from the Tooth Bank of the Estácio de Sá University, Rio de Janeiro, RJ, Brazil. The teeth were extracted for orthodontic or prosthetic reasons. The study was approved by the Ethical Committee at Estácio de Sá University (approval number: 34551214.2.0000.5284). The coronals and the apical thirds of the teeth were removed using diamond disks (KG Sorensen Ind. Com. Ltda, Barueri, Brazil). Thereafter, the middle thirds of the roots were split along the long axis and cut into 25 mm^2^ fragments. The 94 specimens generated were immersed in 2.5% NaOCl solution for 5 min and then in 17% EDTA (Biodinâmica, Ibiporã, PR, Brazil) for 5 min, followed by washing with 2.5% NaOCl for 5 min to remove the smear layer formed by the cutting action of the disks and any pulp tissue remaining. During these procedures, all solutions were agitated in an ultrasonic bath at a frequency of 50 Hz (Cristófoli, Campo Mourão, Brazil). Finally, the root fragments were washed with distilled water and sterilized by autoclaving.

### *E. faecalis* biofilm formation

**Figure 1:**
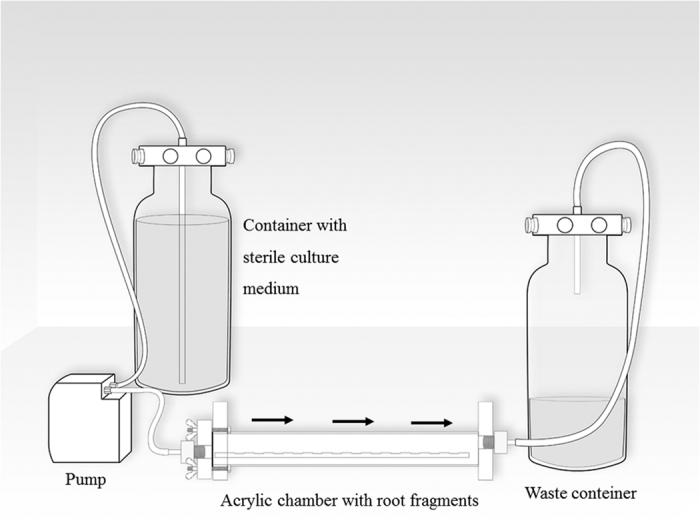
The apparatus used for bacteria incubation. Black arrows indicate the direction of the culture medium flow.

The root fragments were infected with *E. faecalis* (ATCC 29212) using an apparatus described by Luppens^26^ and specially adapted by the authors for the present study (Figure 1). The apparatus is composed of an acrylic chamber, a peristaltic pump (Exatta, Palhoça, SC, Brazil) and two 9-liter glass containers. The three components were connected by silicone tubing to have a constant flow of the culture medium. All components and supports were cleaned with 70% ethanol and autoclaved before use.

Before inoculation, the cementum surfaces of the 94 root fragments were bonded onto the internal acrylic base of the apparatus. Afterwards, the medium Tryptic Soy Broth (TSB, Difco, Detroit, USA) supplemented with 10% glucose (Merck, Whitehouse Station, USA). was pumped through the system for 30 min after which it was removed. Then a 24 h culture (20 ml) of *E. faecalis* was introduced into the device and was maintained in contact with the root fragments for 30 min. After this period, the pump was restarted, and samples were allowed to develop biofilm for 24 h at 37 °C in the presence of a constant TSB flow of 6.25 ml/min. At the end of this 24 h period, the root fragments were removed from the device and placed into cell culture wells (1 dentin block per well) of a 24-well plate (Nest Biotechnology, Wuxi, China). The manipulation of root fragments during the experiment was performed aseptically in a laminar flow hood (Nuaire, Plymouth, MN, USA). The quality control of the materials sterilization process was attested by the Institutional Sterilization Center.

Two samples were used to confirm the biofilm formation. On removal from the device, they were immediately fixed in freshly prepared 2% glutaraldehyde (Merck, Whitehouse Station, NJ, USA) and then dried in ascending ethanol concentrations. They were then dehydrated to their critical point in CO_2_ and sputter-coated with gold under vacuum and analyzed in a scanning electronic microscope at 10.00 Kv and at 5000 magnification (Inspect F-50, FEI, Hillsboro, OR, USA).

### Dentin disinfection assay

The root fragments were divided randomly into 4 groups (NaOCl, CHX, ALX and NaOCl+ALX) of 20 blocks each and 12 samples were separated for the control group. The root fragments of the NaOCl, CHX, and ALX groups were immersed in 1 ml of 2.5% NaOCl, 2% CHX and 1% ALX for 10 min, respectively. The 1% solution of ALX was prepared by dissolving ALX dihydrochloride powder (Sigma-Aldrich, St Louis, MO, USA) in sterile distilled water (1 g/100 ml). The samples of the NaOCl+ALX group were immersed in 1 ml of 2.5% NaOCl for 10 min followed by 1% ALX for 10 min. In all groups, except the control group, a neutralizer solution was used for 5 min after the action of the irrigants. This solution was composed of 3% Tween 80, 0.3% lecithin, 0.1% histidine and 0.5% sodium thiosulfate. In the control group, the root fragments were immersed in 1 ml of sterile saline for 10 min.

Microbial samples were obtained from root fragments by agitation in ultrasound for 3 min. Tenfold serial dilutions were carried out in saline. Then, aliquots of 20 μl of each dilution were plated onto Mitis-Salivarius agar (Difco, Detroit, MI, USA) plates, and incubated at 37° C for 24 h. The colony-forming units (CFU) that grew were counted and then transformed into actual counts based on the known dilution factors.

Bacterial counts were analyzed via Kruskal–Wallis and Mann–Whitney tests. The significance level was established at *P*<0.05. The statistical analysis was performed using SPSS 17.0 computer software (IBM, New York, NY, USA).

## Results

**Figure 2:**
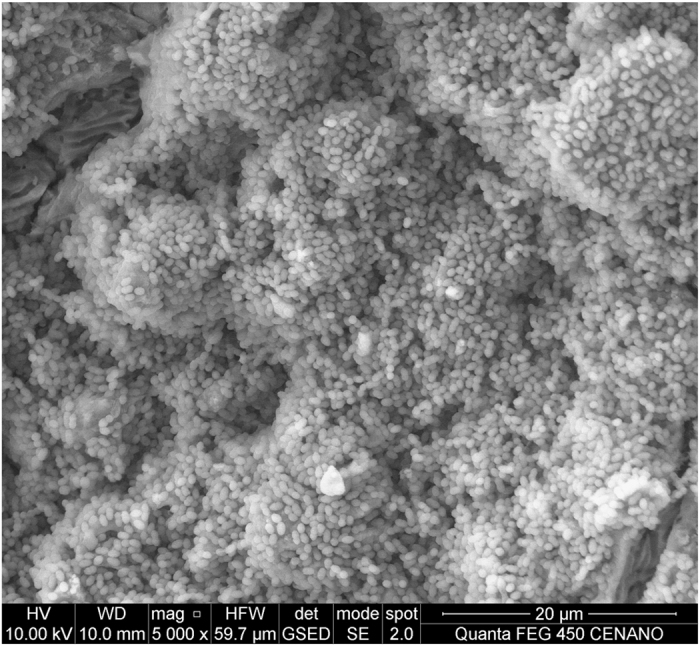
SEM image of a dentin block infected with *E. faecalis* biofilm.

An *E. faecalis* biofilm was observed by electron microscopy on both fragments analyzed (Figure 2). Intergroup analysis revealed no significant difference among the experimental groups (*P*>0.05) except for the comparisons CHX versus ALX, and NaOCl+ALX versus ALX (*P*=0.004). ALX alone was the less effective irrigant. CHX and NaOCl+ALX eradicated all bacterial cells in all samples. The NaOCl group showed bacterial growth only in one of the 20 samples while ALX showed bacterial growth in seven of the 20 samples (Table 1). All experimental groups were significantly more effective than the control group (*P*<0.05).

## Discussion

Biomechanical cleaning with files and antibacterial irrigants reduces the bacteria load in infected root canals; however, microbial communities grown in biofilms are remarkably difficult to eradicate with antimicrobial agents.^27^ There are reports showing that microorganisms grown in biofilms could be 1000–1500 times more resistant to antimicrobials than planktonically grown bacteria.^27,28^ This *in vitro* study compared the antibacterial effect of ALX, a promising root canal irrigant, alone or as a final irrigant in combination with NaOCl, with the most common root canal irrigants: NaOCl and CHX.

*E. faecalis* was chosen as a bacterial marker since its resistance to many intracanal disinfectants is well documented^2,4,29,30^ Gram-positive facultative anaerobe bacterium is commonly found in endodontically treated root canals that failed^2^. The persistence of *E. faecalis* may stem, in part, from its ability to form biofilms in root canals and its capability to invade dentinal tubules.^31,32^ Additionally, this bacterium possesses a plethora of virulence factors, highlighting: aggregation substances, surface adhesins, sex pheromones, lipoteichoic acid, extracellular superoxide, gelatinase, hyaluronidase, and cytolysin (hemolysin).^4^

In the present study, the inoculation apparatus allowed the formation of the biofilm under a slow turbulent flow to facilitate the adhesion of cells. When a tooth undergoes pulpal necrosis and subsequently develops periradicular periodontitis, exudates may cycle in and out of the canal. However, the exact flow rate that occurs *in vivo* has not been determined. This fluid exchange provides proteins, glycoproteins and other nutrients to the bacteria growing as a biofilm. This not only provides a sustainable nutrient source but also exerts a shear force on the bacterial biofilm.^33^

Contrary to expectations ALX alone was the less effective irrigant, but its combination with NaOCl was similar to CHX. Two previous studies compared the antibacterial activity of ALX and CHX, in the same concentration, and neither study found any significant difference. The first tested the canal irrigants against *E. faecalis* infected bovine dentin^34^ and the second compared these irrigants against *Streptococcus mutans* biofilm cultivated on human dentin blocks.^35^ Methodological differences such as the substrate and the bacterium tested could have influenced these results. Contrary to these results, another study^21^ found a better antibacterial substantivity against *E. faecalis* using 1% ALX in comparison to 2% CHX. However, it is important to emphasize that in this substantivity assay, the antimicrobial action was evaluated over a period of 80 days. Also, the dentin fragments were immersed in the antimicrobial solution first and after transferred to the bacterial suspension, which is the opposite sequence from the other studies. In the present and previous studies, the antibacterial action was analyzed only once, immediately after the irrigant contact time.

The results from the present study are in accordance with a recent study^36^, which found that 5.25% NaOCl was highly effective against *E. faecalis* compared with CHX and ALX. There was no significant difference between 1% ALX and 2% CHX. Despite both studies used different concentrations of NaOCl, it is not expected significant differences in the antimicrobial activity of NaOCl varying its concentration.^37–39^

The best results were obtained with 2% CHX and with the combination of 2.5% NaOCl+1% ALX as a final irrigant. In fact, both substances completely destroyed the bacterial biofilms. However, CHX in not able to dissolve organic tissues. Thus, the combination of NaOCl+ALX has a good potential for endodontic treatment to eliminate biofilms: the solvent capability of NaOCl, the high biocompatibility of ALX, the advantage that it does not form any precipitate when in combination with NaOCl and now, the confirmed antibacterial efficacy of the tested protocol, compatible with CHX and NaOCl alone, justify this potential. However, it is important to highlight that the group NaOCl+ALX was privileged by a higher contact time between the root fragments and irrigant solutions (20 min) in comparison with the other groups (10 min). This difference was necessary since ALX was used in this group as a final irrigant. Certainly, further studies are required to compare this final irrigation protocol with others.

Under the conditions of the present study, it was concluded that 1% ALX alone should not be indicated as an intracanal irrigant since its antibacterial effect against *E. faecalis* was inferior to 2% CHX and 2.5% NaOCl. However, the combination of NaOCl with ALX as a final irrigant has potential to be used in endodontic treatment to eliminate biofilms.

## Publisher’s note

Springer Nature remains neutral with regard to jurisdictional claims in published maps and institutional affiliations.

## Figures and Tables

**Table 1:** *Enterococcus faecalis* counts after treatment with the tested irrigants

Groups	*N*	Samples showing growth	Median	Mean	Range
2.5% NaOCl	20	1	0	0.8×10	0–1.60×10^2^
2% CHX	20	0	0	0	0
1% ALX	20	7	0	1.94×10^2^	0–1.25×10^3^
2.5% NaOCl+1% ALX	20	0	0	0	0
Saline	12	12	4.25×10^4^	8.96×10^4^	2.05×10^2^–4.40×10^5^

Abbreviation: CFU, colony-forming units.
